# A Stick of Candy

**Published:** 1870-01

**Authors:** 


### A STICK OF CANDY.

We have too lively a recollection of the delights of taffy and sugar-stick to embitter these sweets, so dear to juveniles, with any unnecessary questioning of their innocence. It may be well, however, to state simply the scientific facts in regard to candy and its fellow-compounds of sugar, in order that the discretion of our youthful friends, being duly guided, shall be able to moderate their excessive proclivities to pulled molasses and chocolate drops.

Sugar, which is the chief constituent of the various sweets in which the youthful frequenter of the confectionary shops is wont to indulge, is undoubtedly a nutritious article of diet. It exists abundantly in vegetable food, forming a part of all the fruits and grains, and enters largely into the composition of some animal substances, especially milk. The blood of animals contains a notable part; and a German Chemist pretends to have discovered in the muscular tissue something with the identical chemical, though not physical, characters of sugar. A sugar-house, in fact, forms a part of every human structure, where this sweet substance is made in order to supply the natural wants of the living being. The liver, among its other functions, converts the albuminous matters which pass through it into sugar. The blood thus furnished has a constant supply quite independent of the saccharine matter contained in the food eaten. Liebig thought that the only use of sugar in the system was to serve as fuel to keep up combustion, and thus aid in supplying the due quantity of animal heat; but as there are many insects which feed solely on this substance, it is more logical to suppose that it is essential in some way to organization.

There is a popular notion, although it does not seem to check the general consumption of sugar, that it is injurious to the teeth. The brilliant ivories set between the thick, ruddy lips of the West Indian negro, who is an inordinate devourer of sweets, would seem to contradict this. There are some persons, however who, from some derangement of their functions of digestion, can not eat sugar without its turning at once into acid, which, undoubtedly, among its other evil effects, will corrode the teeth.

Sugar, when pure and used in moderation, is not only not injurious, but a wholesome and necessary article of food. Parents, therefore, may mitigate their anxiety, if not in regard to the moral, at least the physical effects of the frequent larcenies by their little ones of the sugar-bowl, with the reflection that these stolen sweets are not very dangerous after all. Pure unsophisticated candy may also be moderately indulged in without risk. The simple sugar-sticks and chocolate cakes are less likely to be injurious than any painted or perfumed confection.

The consumption of saccharine compounds does mischief not so much from any direct ill effect they may produce as by being eaten at untimely seasons, and in such excess as to exhaust the appetite, and thus create an indispotion for the regular meal and more nutricious food. We have no doubt that much harm thus results: for the habit is common, especially with children and women, of frequenting the confectionery shops during the period of the usual promenade which precede the main repast of the day, and when the stomach is so eager to devour that it is almost impossible to check its appetite for whatever may be offered to it. It is then that the palatable but not very nutritious *bonbons* and other saccharine frippery are swallowed voraciously, and preoccupy the space which should have been kept for solid beef and pudding. Sweets should always be held in reserve and in subordination as a dessert to plain food, for their taste is so decided and all-absorbing that they soon satiate and indispose the palate for anything else. There is no good reason why little children, and those of larger growth, should not, if at the proper time and it be of moderate length, indulge their liking for *a stick of candy*.—*Harper's Bazaar.*

